# One Year Probationers

**Published:** 1887-04-30

**Authors:** 


### ONE-YEAR PROBATIONERS.

The authorities of the North London or University College Hospital, state that "there is a mistake in Miss Landell's information with regard to 'One-year Probationers' in this hospital. Any who come only for training, whether for one year or for a shorter period, pay 12*s*. per week, exclusive of washing. We are sending you a report of our Nurses' Home, which will give you all information as to our system of nursing. The Hospital Committee pay the All Saints sisters and nurses a certain sum annually, and engage to supply the hospital with nurses."

